# Supplementary material for: Modelling sensory attenuation as Bayesian causal inference across two datasets
Source: PLoS One. 2025 Jan 24;20(1):e0317924. doi: 10.1371/journal.pone.0317924 (PMC11761661; doi:10.1371/journal.pone.0317924)
Supplement: S1 File — Supplementary materials and analyses on belief propagation, parameter recovery, outliers, effect of T. (DOCX) [file pone.0317924.s001.docx]

Supplementary Materials.

**S1.** Belief propagation in graphical networks.

Graphical networks are useful tools to efficiently compute marginal probability distributions. In the present work, we are interested in state inference on the basis of noisy sensory observations. Specifically, for Experiment 1, participants need to infer the cause of a tactile frequency presented to their fingers, and in Experiment 2, participants need to detect the common cause (or the separate causes) of proprioceptive and visual information. Responses are in two-alternative-forced choice (2AFC) format (Experiment 1: probe/ no probe, Experiment 2: delay/ no delay), on the basis of which we simulate the underlying dynamics of evidence accumulation and decision-making using belief propagation.

We develop Bayesian graphical networks, where the unobservable nodes (states) form a Markov chain with one observable node attached. We are interested in the marginal probabilities of each possible state (Experiment 1: cause $\left\{ internal, external, none \right\},$ Experiment 2: arm position $\left\{ 0,1 \right\}$). To obtain the marginal probabilities for all states in a variable node $V$, the product of all incoming messages from neighboring factor nodes is computed:

|  | $p\left( V \right)= \prod_{l\in ne(V)} \mu_{F_{l\to V}}(V)$ | [S6] |
| --- | --- | --- |

where $ne(V)$ are all neighbors of the variable node $V$, $\mu_{F_{l\to V}}$ is the message received from factor node $F_{l}.$The factor graph is bipartite and messages can only be passed from variable- to factor nodes and vice versa. A message from factor- to variable nodes $\mu_{F\to V}\left( V \right)$ is given by

|  | $\mu_{F\to V}\left( V \right)= \sum_{V1} \ldots\sum_{V_{M}} F\left( V_{1}, V_{2}, \ldots V_{M} \right)\prod_{m\epsilon ne(F)\backslash V} \mu_{V_{m}\to F}(V_{m})$ | [S7] |
| --- | --- | --- |

All messages the factor node has received from the neighboring variable nodes $\mu_{V_{m}\to F}(V_{m})$ (except the recipient variable node $V; m \epsilon ne(F)\backslash V$) are multiplied with the factor and summed over all variables except $V$. When the recipient variable is the factor node’s only neighbor (i.e. $ne\left( F \right)\backslash V=0$), the factor information is sent.

|  | $\mu_{F\to V}\left( V \right)=F(V)$ | [S8] |
| --- | --- | --- |

Messages from variable- to factor nodes take the form

|  | $\mu_{V\to F}\left( V \right)= \prod_{l\epsilon ne(V)\backslash F} \mu_{Fl}\to V$ | [S9] |
| --- | --- | --- |

Variable node $V$ forms the product of all received messages from neighboring factor nodes except the recipient factor node $F$. When $F$ is the only neighbor of $V$, the message reduces to

|  | $\mu_{V\to F}\left( V \right)=1$ | [S10] |
| --- | --- | --- |

For unobserved variable nodes, or the respective observation if $V$ is observed.

**S2.** Parameter recovery

We performed a prediction recovery to validate the model for both experimental datasets.

Experiment 1.

**Prediction recovery**. The goal of the prediction recovery is to assess whether our fitting procedure is capable of capturing ground-truth model predictions of probe detections, $p(ext).$ The general procedure involves testing the impact of varying individual model parameters on model-predicted detection probabilities, $p(ext)$, and its relationship with simulated response frequencies and recovered detection probabilities $(p({ext}_{M}))$. A close overlap between generating and recovered $p(ext)$ with simulated response frequencies indicates successful recovery of model predictions and indicates that the fitting procedure did not introduce systematic biases.

Each model parameter (here, the four parameters that determine transition dynamics, $p(ext|int), p(ext|ext), p(int|ext), p(int|int)$) was individually and randomly initialized, while all other model parameters were held constant at the value that resulted from the optimization procedure. With this, we can establish the effect of randomly varying the parameter of interest on simulated $p(ext)$. Each parameter was randomly initiated 50 times. There were some endpoint constraints on the time series, which we enforced via rejection sampling. We discarded a parameter sample if the resulting model violated $p(M_{5}=None) \sim1$ at the beginning of the time series (before any stimulus was presented), and $p(M_{40}=int) \sim1$ at the end of the time series (i.e., after object contact). Parameter sets who could not conform to these constraints were not considered in our prediction recovery experiments. When a parameter set was found that fell within these constraints, the model was then fit to obtain the recovered, model-based detection probability. The model fitting was performed with similar settings as described in the main manuscript, except for holding the priors on the first state of the timeseries at uninformative levels, since they did not show systematic relationships with model-based detection probability.

Results are shown Figure S15. Our fitting procedure allows for a tight recovery of predictions. Simulated detection probabilities are driven by the ground truth data in the expected ways. This is particularly true for parameter $p(ext|ext) \& p(ext|int)$ for incongruent trial types, where a larger range of generating detection probabilities can be recovered. The reason for this is driven by the nature of the data in each trial type: the stability of the external cause is particularly important for reliably detecting the probe in incongruent trials, as is the transition to an external state from an internal state. In congruent trials, the stability of an external cause is not relevant, because the model predicts the external state – if it occurs at all – to be very instable across different trials of the same, congruent type. The model hence should predict a low $p(ext)$ for congruent trials, which is reflected in panels **S15A & B**. The transitions into- and away from an external state are well recoverable in incongruent trial data. Not also that most parameters fall within a tight range of possible values when our endpoint constraints on $p\left( M_{t}=None \right),p\left( M_{t}=internal \right)$ are in place. These constraints reduce the degrees of freedom in our model and therefore prevent overfitting. The recovery suggests that as long as all other transition parameters lie within a certain range, the model produces behavior consistent with the training data.

The endpoint constraints on the time series make a full recovery, where all parameters are varied randomly, impractical, since the parameters need to fall within a certain range in order to be able to reproduce the empirical behavior. For a full parameter recovery, our model would require less sparse data that is predictive for every possible state transition, and sample participants causal inference at several points throughout the time series.


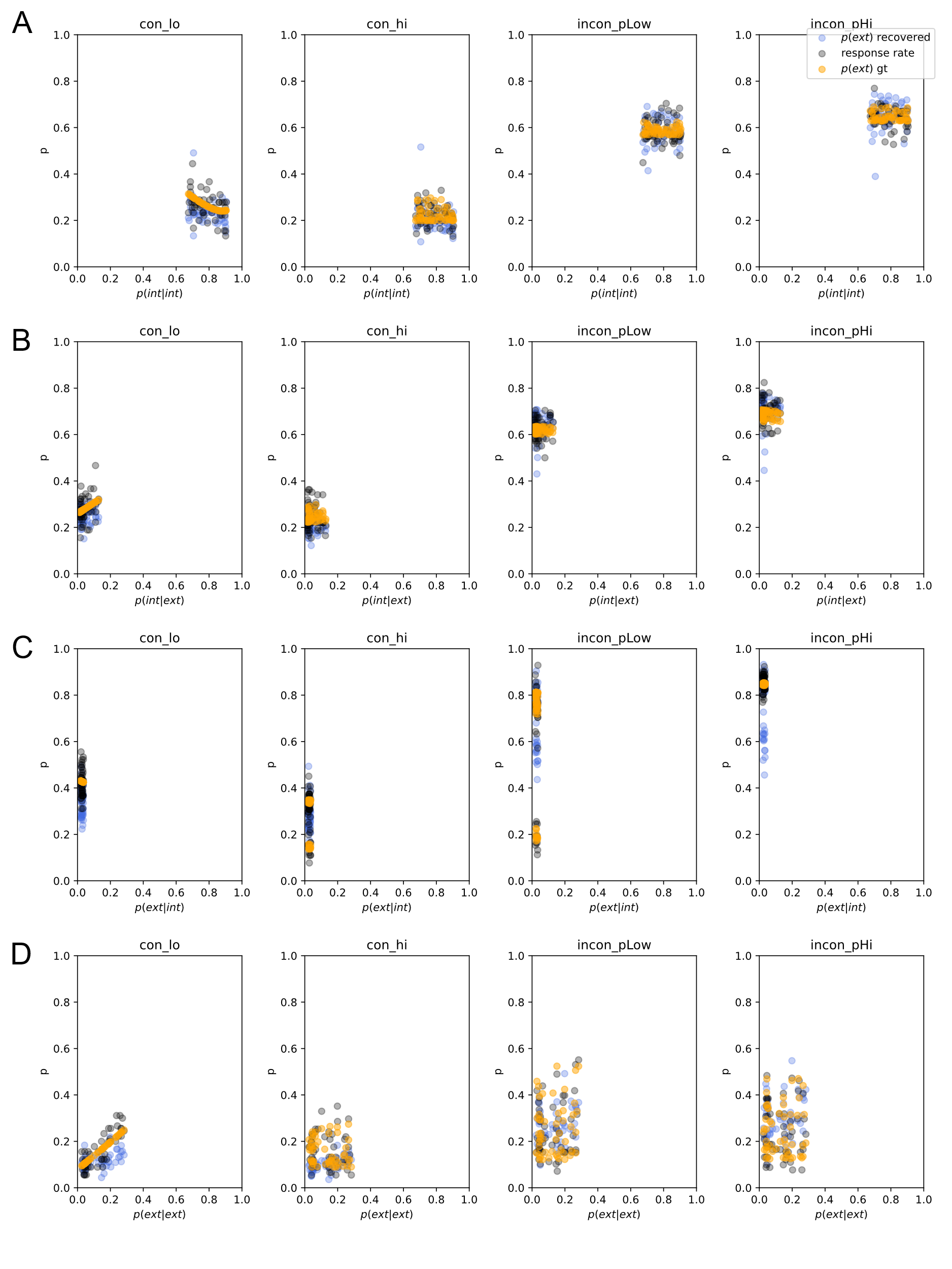


*Figure S15. Prediction recovery, Exp. 1*

**A.** Recoverability of transition parameters in congruent-low trials **B.** Congruent – high trials **C.** Incongruent trials, probe low, object high frequency. **D.** Incongruent trials, probe high, object low frequency.

Experiment 2.

The procedure for prediction recovery followed a similar logic to the procedure described above for Experiment 1, beginning with investigating the impact of each model parameter on simulated responses ($p(separate causes), p(sep)$) and the generating probability of a detection response.

1. Initialize the model with constant parameters, except for the parameter of interest, which is initialized with random values.
2. Simulate 100 trials with these values and obtain average response probabilities per delay.
3. Repeat 50 times for each parameter.

Finally, to obtain the recovered detection probabilities, we fit the model to the simulated data with optimizer settings equivalent to those described in the main manuscript.

Results are shown in Figure S16 and Figure S17. Except for the prior on the separate-causes chain (Figure S17, row 3, sp_e_0), all parameters show systematic effects on the simulated $p(sep)$ and average response frequencies in at least one delay condition. Generally, the overlap between the simulated detection probabilities and the response frequencies is tight, indicating the procedure worked as intended and ground truth quantities are captured well.


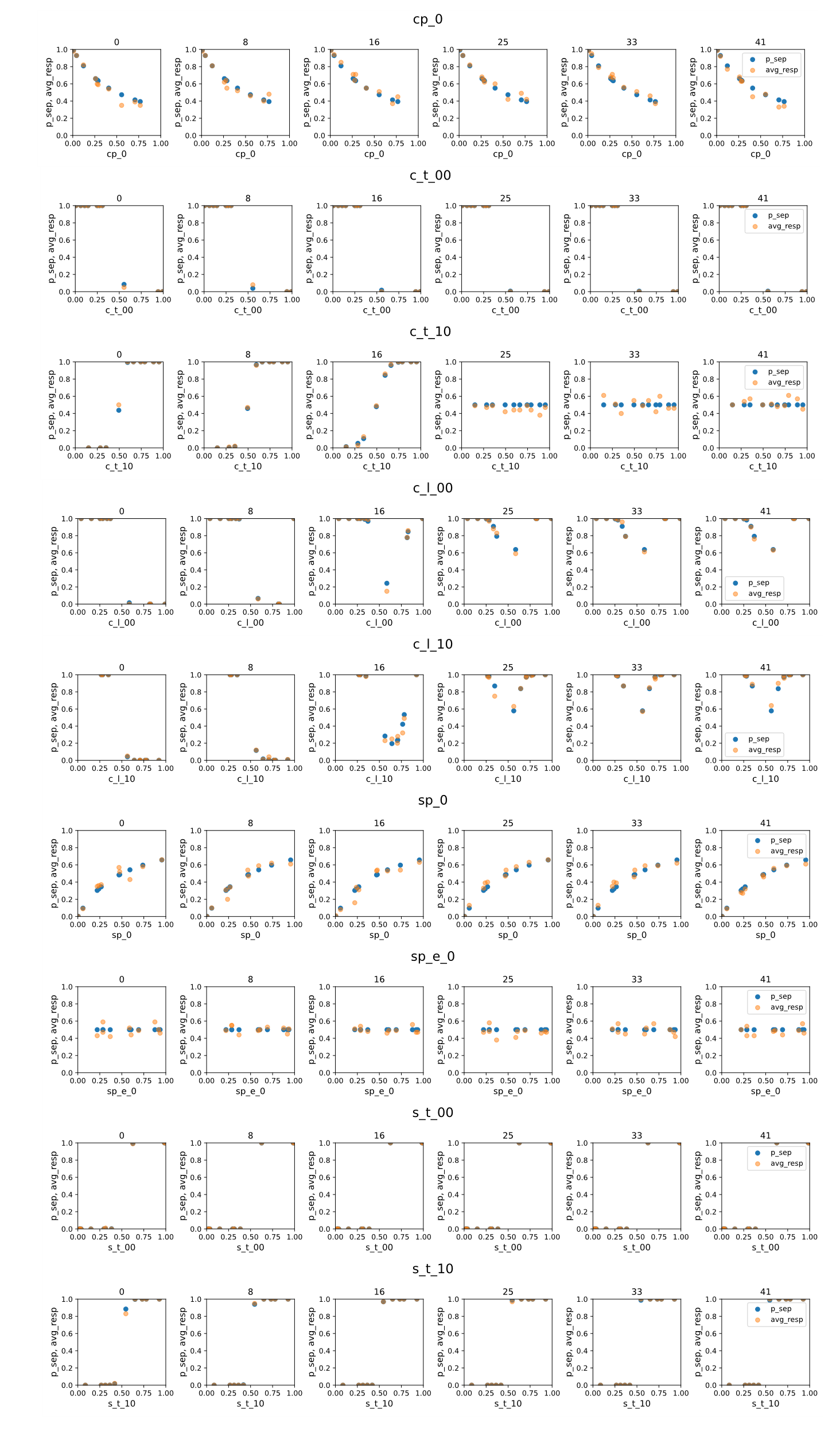


*Figure S16. Prediction recovery, Exp. 2*

Each row represents the randomly initiated parameter, all other model parameters were held constant at the best average fitted values. Each column represents the delay used for the simulation, from 0 to 41ds. Simulated average response probability are scattered against parameter value in blue. Average simulated response frequencies are plotted against parameter values in orange.


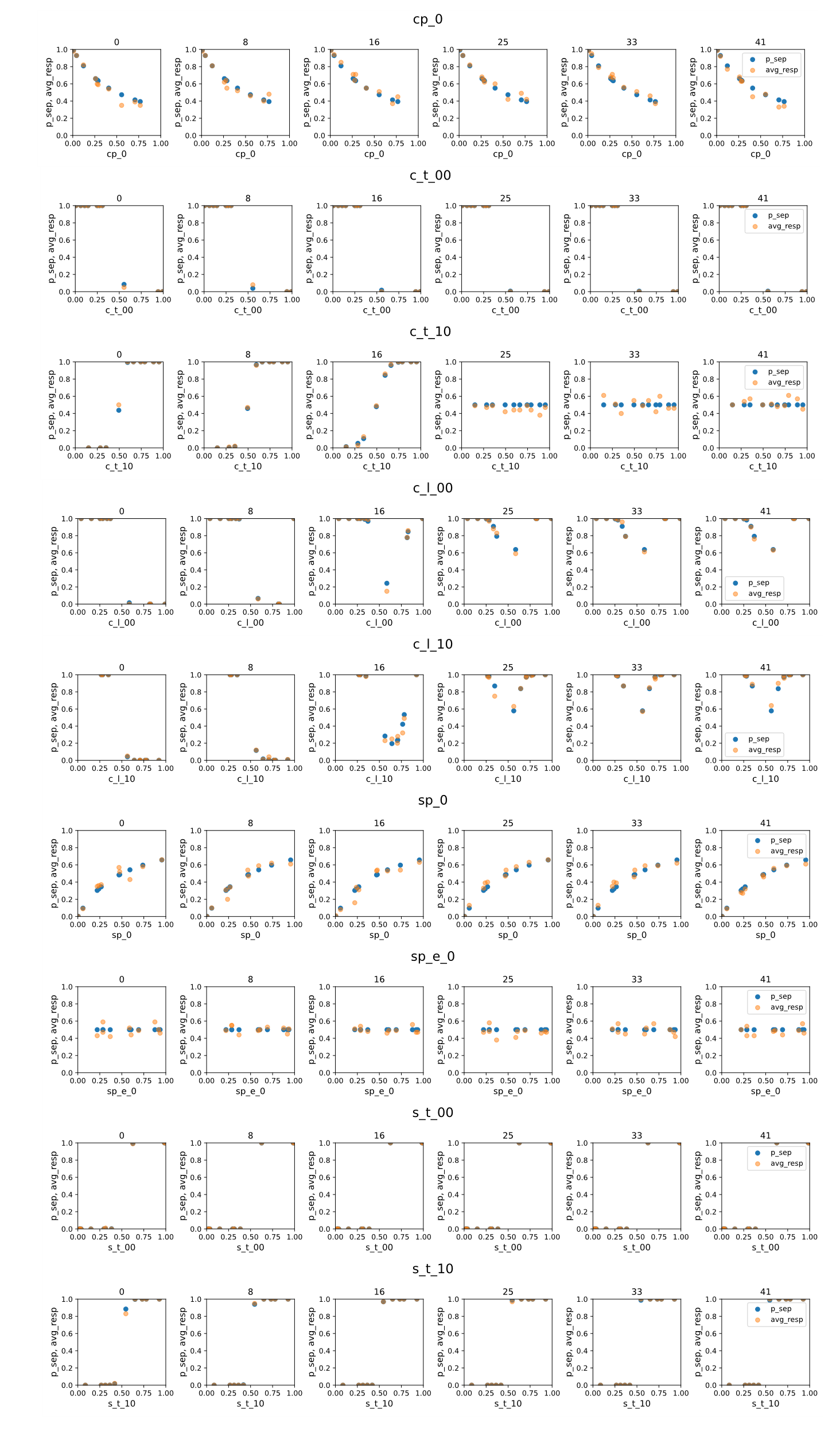


*Figure S17. Single parameter effects on model-derived response probabilities (ctd.)*

Parameters for the separate-causes chain. As before, model-derived response probabilities and average response frequencies are plotted against the respective parameter value in blue and orange, respectively.

**S3.** Outlier analysis

**Experiment 1.** One participant shows a significantly larger KL divergence between model predictions and data (participant nr. 4, KL=12.568 nats). This can be traced back to the lack of variance in their behavior, particularly in the incongruent – probe low condition.


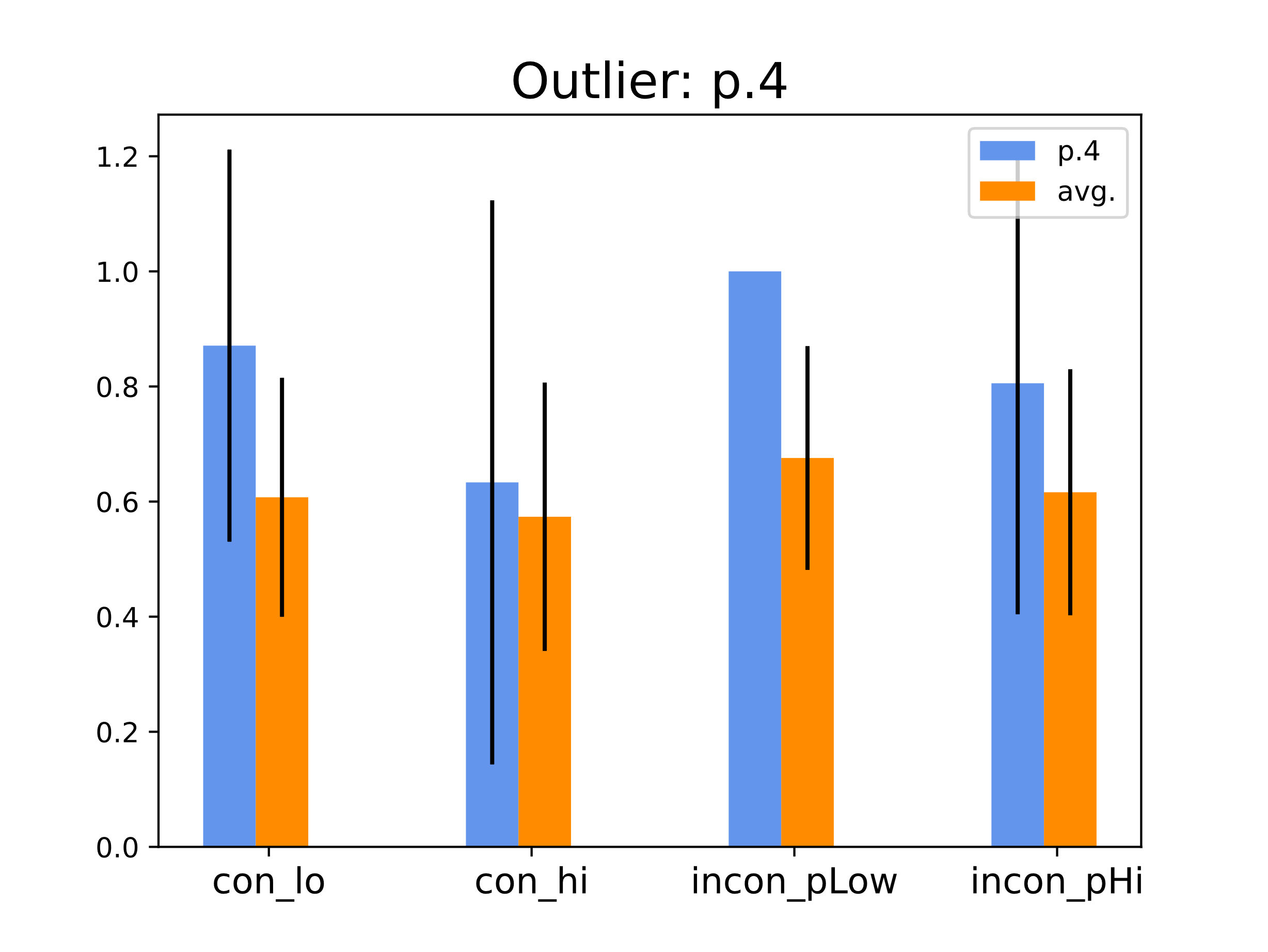


*Figure 18. Outlier participant, Experiment 1.*

**Experiment 2.** Two participants are considered outliers for the fit results of Experiment 2 (P.10: KL=13.267 nats, P.24: KL=10.484 nats). The below figure reveals that these patients had significantly showed significantly lower frequencies of detection compared to average, resulting in poorer model fit.


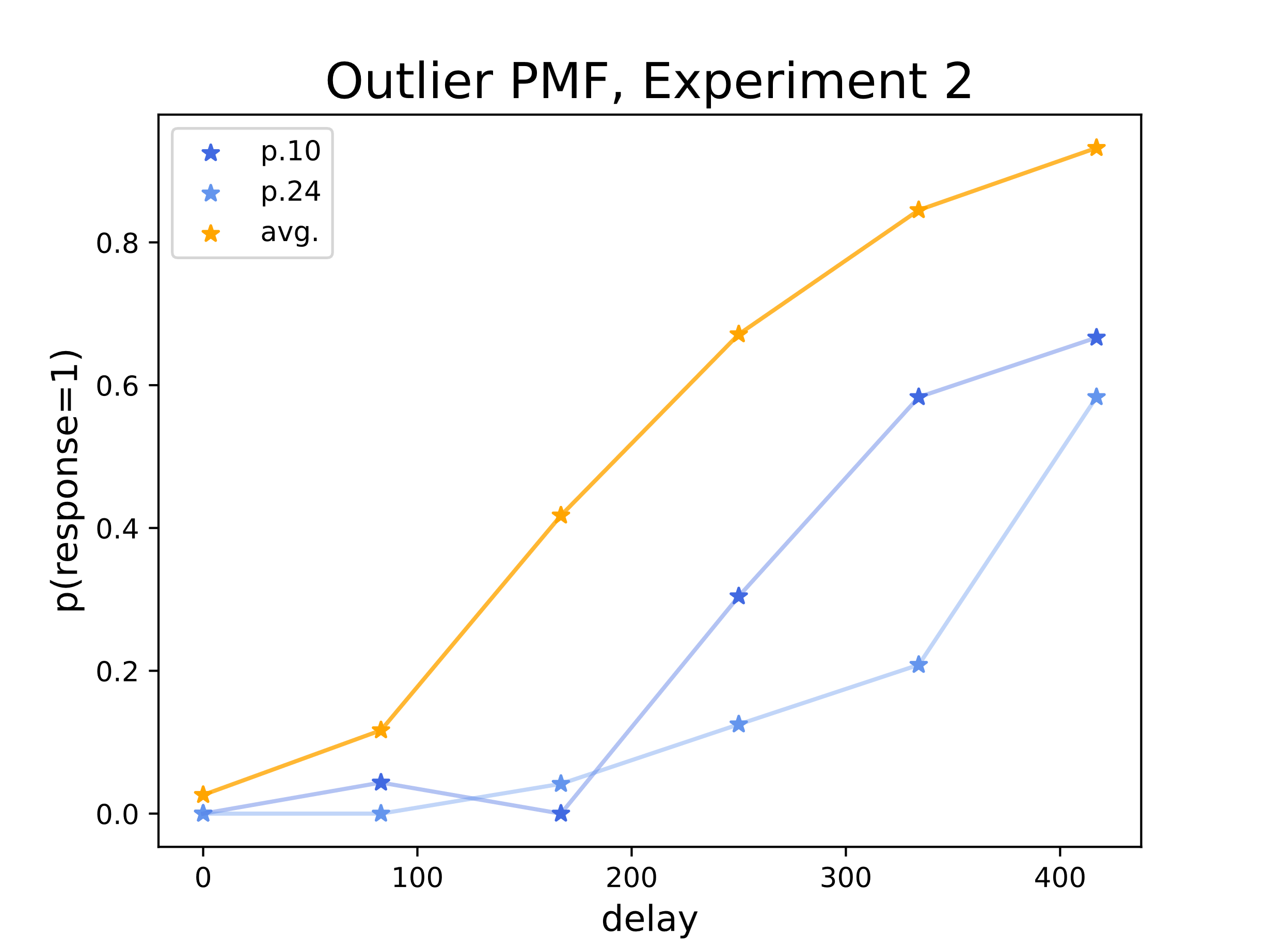


*Figure 19. Outlier participant, Experiment 2.*

In blue shades are the psychometric function of the two outlier participants, where model fits showed a significantly larger KL divergence than average. The orange function represents the sample average psychometric function. The two outliers showed a globally reduced detection performance.

**S4**. Effect of $T$

We investigated the effect of the choice of $T$ (length of the time series) for both experiments. Both for Experiment 1 and 2, the choice of $T$ was based on the duration of the trial in the respective experiment (50). We here test two alternative models for both experiments with a timeseries length of $T=10$and $T=100$, respectively.

*Experiment 1.* The core finding of an increased detection probability for incongruent vs. congruent trials could be reproduced for the short time series with T=10 (see Figure S18, Figure S20) and for a long time series with T=100 (see figure Figure S196, 17). However, the experiment-derived $T$ captured the empirical effects best. With a short model, the temporal resolution might be too low to pick up on the very small behavioral effects within different types of congruent trials. However, there is not a significant benefit of increasing $T$ on model performance. In conclusion, the choice of $T$ closest to the actual experimental conditions might be an important consideration to make when setting up the model.

*Experiment 2.* We attempted to reproduce the core finding of shifted detection probabilities (or psychometric functions) between the active- and passive movement conditions with a model with T=10 and a model with T= 100. In comparison, the experiment-derived initial length of the timeseries was set to T=50. In a short model, the results could not be reproduced, with the model making uninformative predictions (Figure S22). In a longer time series, the quality of the predictions was similarly deteriorated (Figure S23)


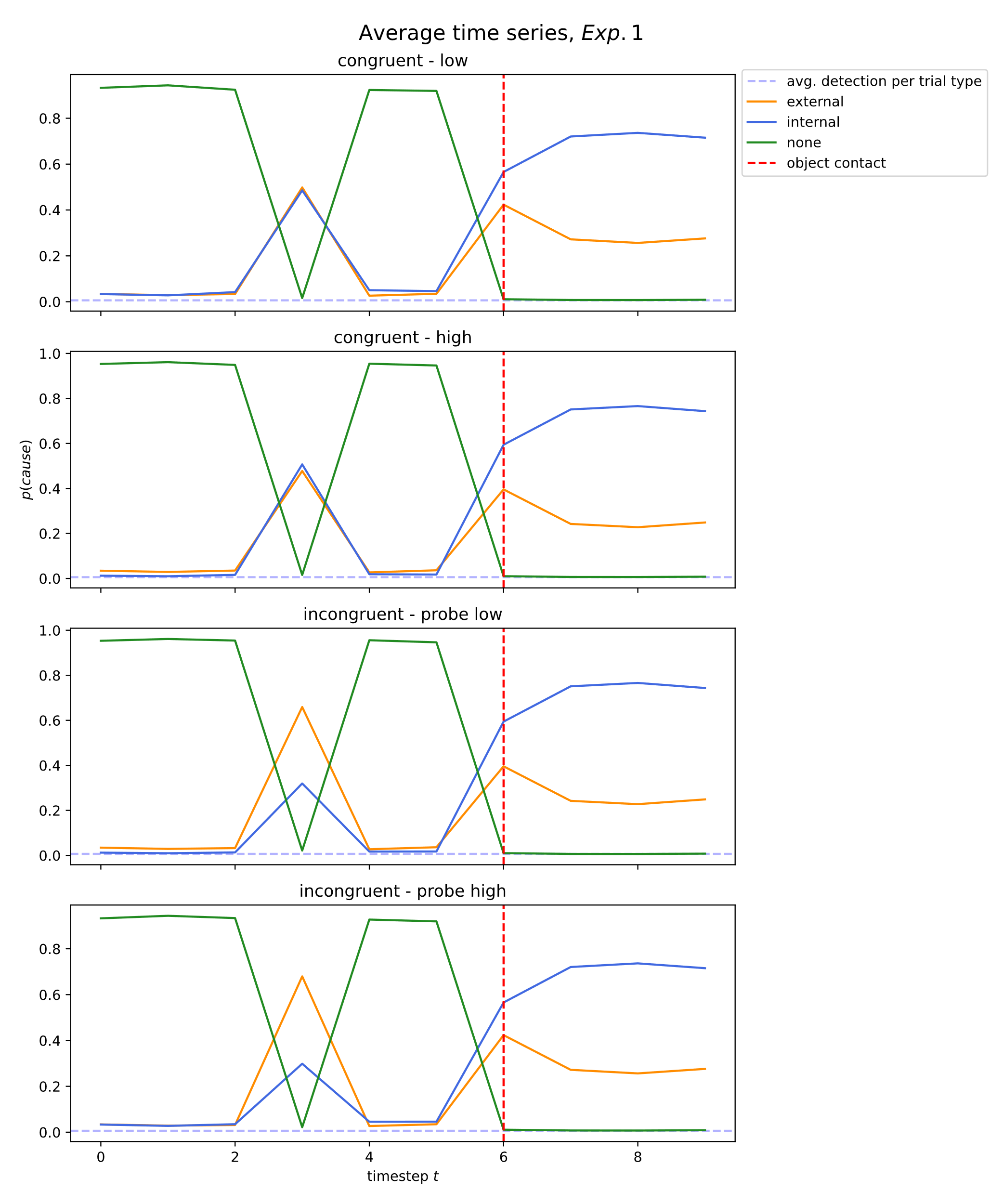


*Figure S18. Results for a model with T=10, Experiment 1*


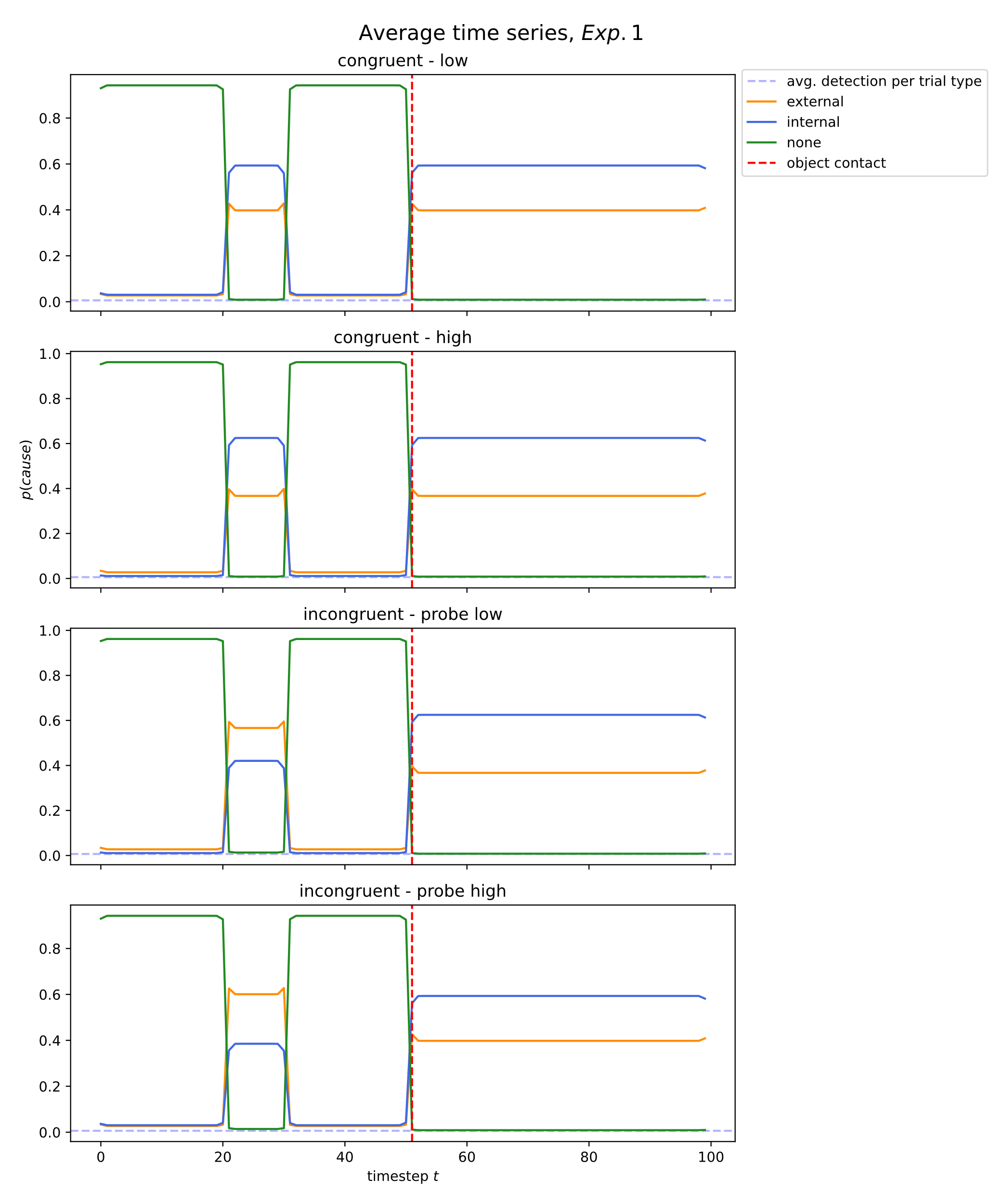


*Figure S19. Results for a model with T=100, Experiment 1*

*
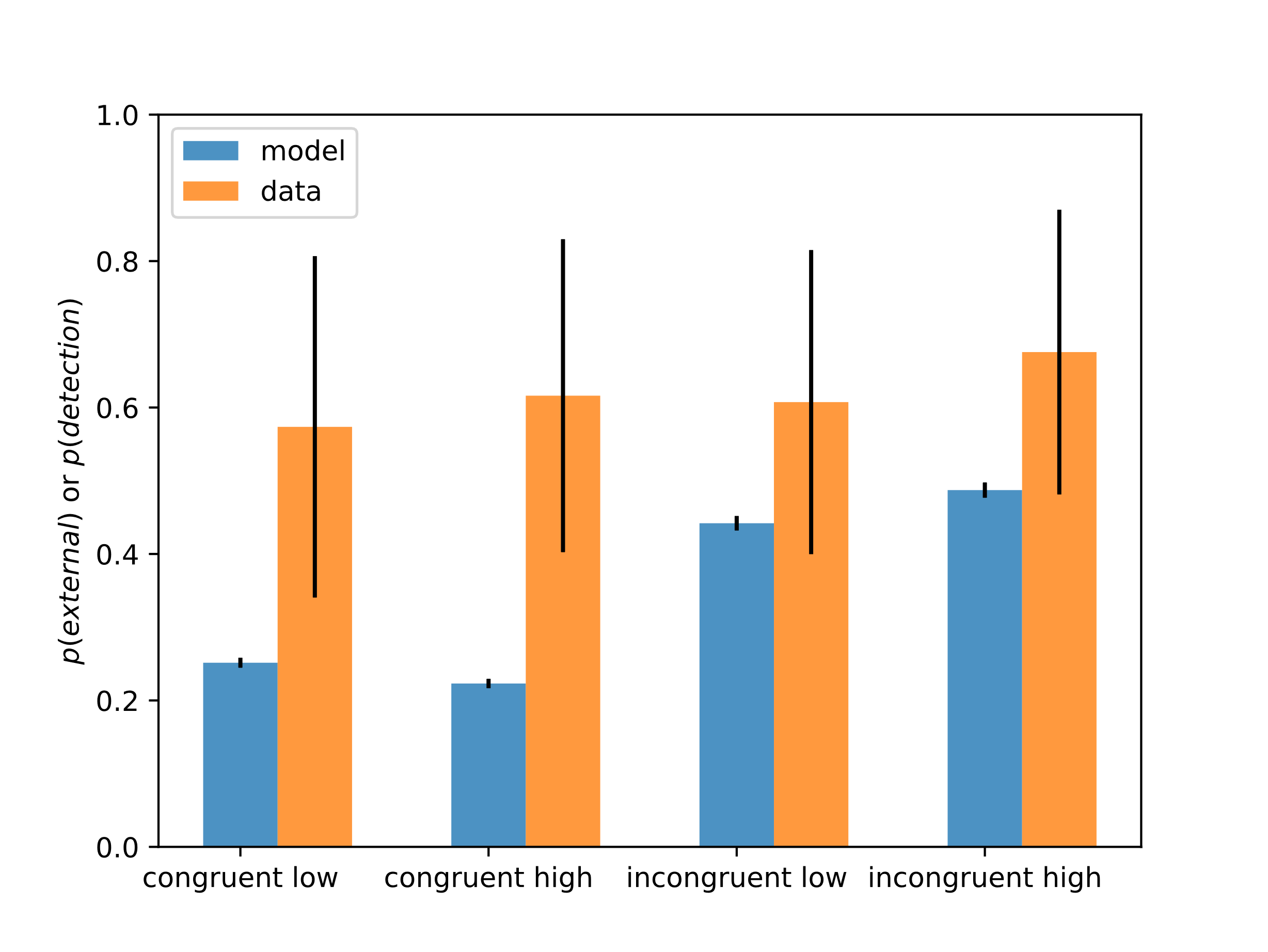
*

*Figure S20. Empirical data vs. model prediction, short series (T=10), Experiment 1.*

*
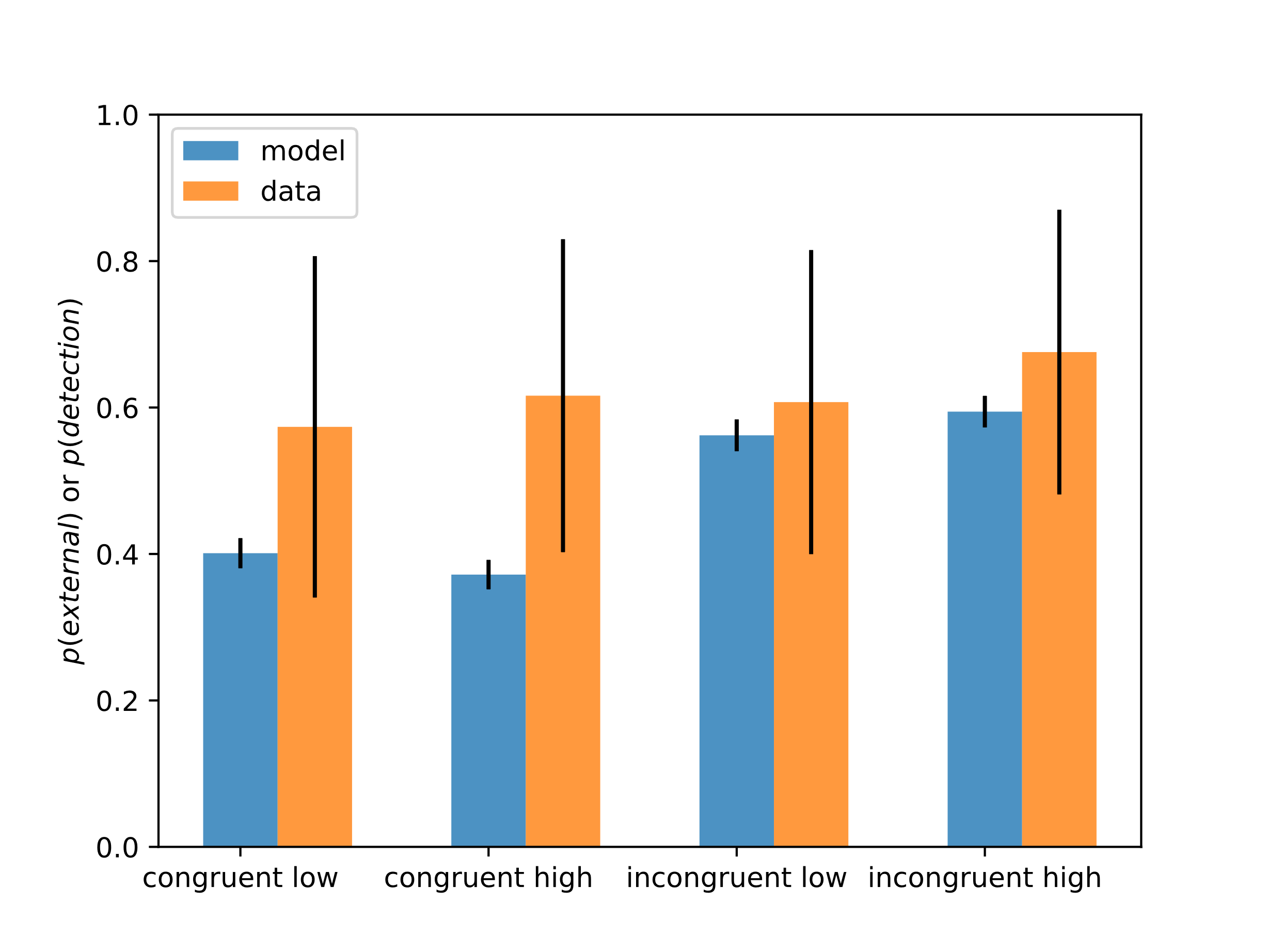
*

*Figure S21. Empirical data vs. model prediction, long series (T=100), Experiment 1.*

*
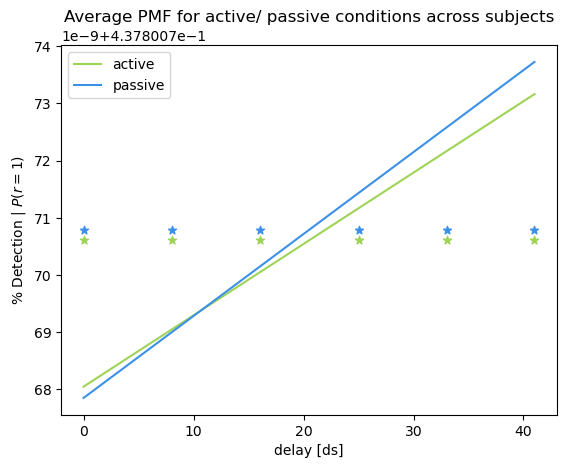
*

*Figure S22 Results for a model with T=10, Experiment 2.*


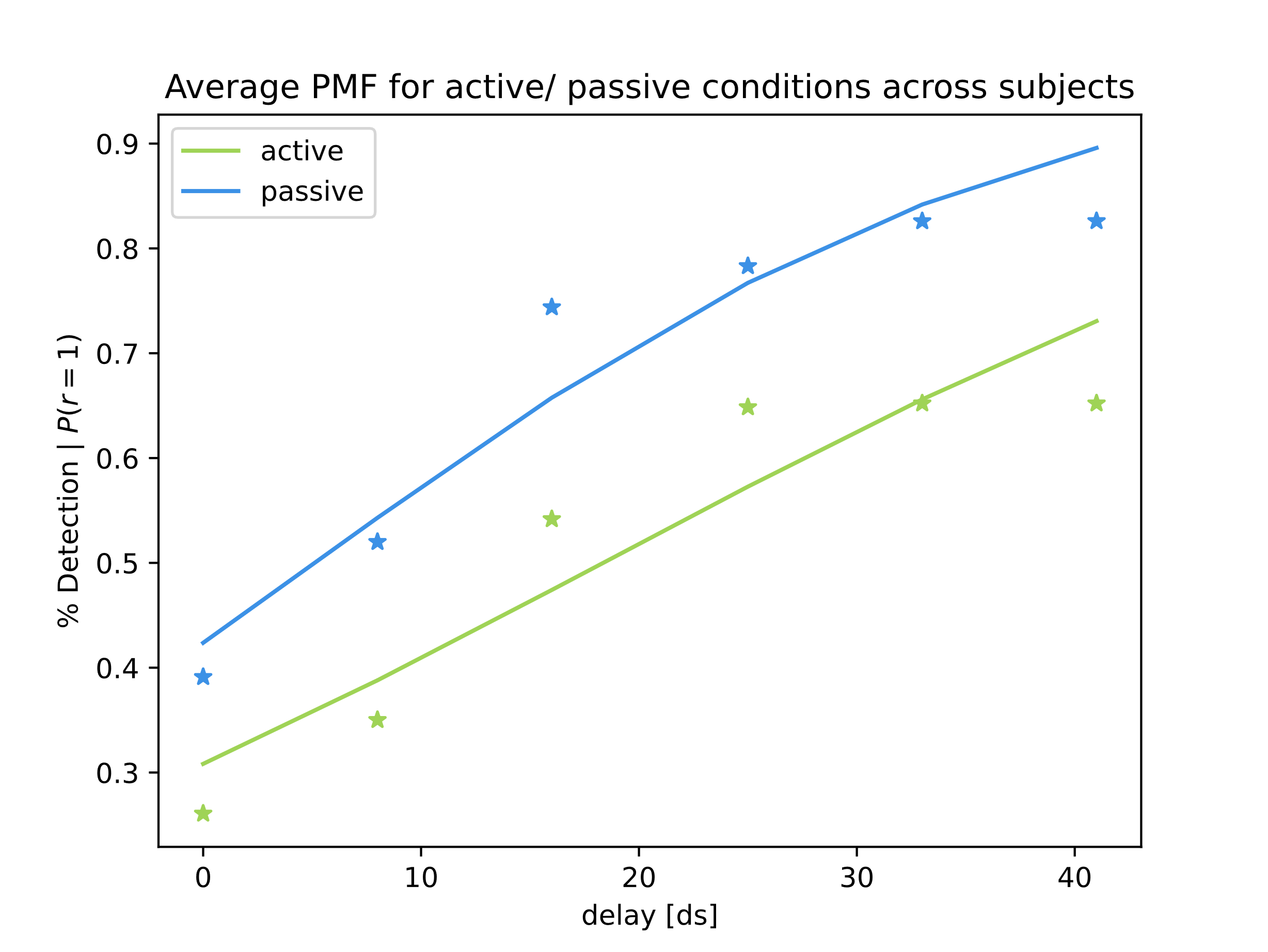


*Figure S23 Results for a model with T=100, Experiment 2.*
