# Supplementary material for: Modelling sensory attenuation as Bayesian causal inference across two datasets
Source: PLoS One. 2025 Jan 24;20(1):e0317924. doi: 10.1371/journal.pone.0317924 (PMC11761661; doi:10.1371/journal.pone.0317924)
Supplement: Attachment — Submitted filename: rebuttal_letter.docx [file pone.0317924.s002.docx]

**Note**: We write the original reviewer comment first, followed by our response (indented with bullet point). Whenever figures were changed, we paste them from the manuscript. Direct quotes of updated text are in *italics.* References to line numbers are highlighted and refer to the manuscript version without track changes (manuscript_rev1_final.doc).

**Reviewer 1**

Reviewer 1, point 1 (**R1.1**)

In order to explain sensory attenuation phenomenae, the authors of this stude develop a model based on the Bayesian Causal Inference model, that infers whether a signal was self generated or external. Specifically they model two previously published experiments, showing that the model can at least qualitatively explain some of the observed effects. This is a technically challenging model, and I appreciate the efforts that have gone into this. The paper is well written, although given the complexity of the model I do worry how much can be learned from it (see more below).

- Thank you for your positive words on our work.

**R1.2**

Models for both experiments have a lot of free parameters to fit (even 12 is a lot). I would be very worried about the model being over specified, hence over fitted. I would be very curious to see a parameter recovery excercise. I.e. if simulated data were drawn from a model with a specific set of parameters, how well could these parameters be recovered when doing the model fitting as in the manuscript (but now to simulated data for which you know the true parameters). Even if the fitted parameters are not of interest, there would always be a worry that the model is not addressing a real aspect of the data, and that any sufficiently detailed model (with e.g. 20 parameters) would have been able to capture the data. I realise that doing more simulations would be time consuming, but I think the authors should find a way to address this concern.

- The reviewer is right in suggesting that a parameter recovery is important for validating our model. We have performed a parameter recovery and added our insights into the revised version of the manuscript.
- The parameter recovery for the two experiments followed a similar logic:
  - **Experiment 1**:

1. initialize subject-specific model with best parameters obtained from the optimization, and compute probability for the hidden causes (external, internal, none).
2. Make a weighted random choice between 1 (detection of probe) and 0 (no detection), where the weights are provided by the model-predicted p(external) (for a 1 response) and the model-predicted p(internal)+p(none) (for a 0 response).
3. Build dataset with sampled responses
4. Perform optimization on the sampled data per participant and obtain subject-specific parameters based on sampled data
5. Compare the subject-specific parameters optimized on sampled data with the subject-specific parameters obtained from the optimization on the empirical data.
   - **Experiment 2:**
1. Initialize subject-specific model with parameters obtained from optimization on empirical data and compute marginal probability of a common vs. separate cause model.
2. Make a weighted random choice based on the marginal probabilities of each model, where a “1” choice refers to a delay detection and is weighted by the marginal probability of separate causes, and a “0” represents a non-detection and is weighted by the marginal probability of the common cause model.
3. Build dataset with sampled responses
4. Perform optimization on the sampled data per participant and obtain subject-specific parameters based on sampled data
5. Compare the subject-specific parameters optimized on sampled data with the subject-specific parameters obtained from the optimization on the empirical data.

- Results for Experiment 1 indicate that the parameters can be recovered using this procedure:

Figure S14. Parameter recovery, Experiment 1.

**A.** Empirical- and simulated parameters for Experiment 1 data. Black distributions represent group-level, averaged parameters across 5 runs of the optimization on simulated data. Blue stars represent the mean (with vertical error bars), group-level parameters obtained from optimization on empirical data.

**B.** Motor noise parameter, obtained from empirical data (blue distribution) vs. from simulated data (red distribution).

- These results are mentioned in the main part of the manuscript and in more detail in the supplementary materials S3 (p. 44).
- L. 401: *Finally, the parameters obtained from optimization on empirical data could be recovered on simulated data (see Figure S14).*
- For Experiment 2: p. 49, l. 913

Figure S15. Parameter recovery, Experiment 2.

**A.** Parameter recovery for active trials of Experiment 2. Grey violins are recovered parameter distributions, blue stars represent the mean ground truth parameter, and blue lines are standard deviations. **B.** Parameter recovery for passive trials of Experiment 2.

**R1.3**

How sensitive is the models to parameter choices? E.g number/length of time steps

- To explore this question, we re-instantiated the models for both Exp. 1 and 2 with T=10 and T=100 timesteps, respectively.
- We could show that it might be crucial to select a model duration that is as close as possible to the experimental set-up. There was no added benefit of doubling the length of the timeseries (i.e., doubling the duration of the time series) with respect to the model’s performance.
- **Exp. 1**: Longer- and shorter timeseries could capture the result on a qualitative level, but the quality of the model’s prediction was reduced compared to a model with T=50.
- Short series, T=10, Experiment 1: Long series, T=100, Experiment 1:
-

- **Exp. 2**: While longer timeseries were able to capture the qualitative results, a shorter model was not.
- Short time series, T=10, Experiment 2: Long time series, T=100, Experiment 2:
-

- In conclusion, it might be important to stay consistent with the experimental task when setting up the model.
- All supplementary analyses are presented in the supplementary materials, Figures S17-S22.

**R1.4**

What is the actual transition distribution described in the methods for Exp 1? I could not find it. Is it just a 2x2 transition matrix? If so it should be mentioned in Methods

- We now include a more detailed visualization of our graphical model in the article, including a visualization of the parameters within a factorized representation of the graph, and the joint probability distributions of a given graph. We furthermore provide more details on how the model’s quantities are related to behavioral responses in this figure.
- Figure 2, l. 256

***A.*** *Graphical model for Experiment 1. Hidden nodes* $M_{t}$ *(empty circles) form a Markov chain that can take on values {internal, external, none}. Observable nodes* $F_{t}$ *(filled circles) can process frequency inputs between 0-100 Hz. The joint probability distribution over the network is given by the prior and the product of all transition- and emission nodes. A detection response (*$r_{t}=1$*) occurs when the normalized posterior probability of the external cause exceeds the normalized posterior probability of an internal or none cause. E=external, I=internal, N=none.*

***B.*** *Graphical model for Experiment 2. Under a common cause model (left box), proprioceptive* $P_{t}$ *and visual information* $V_{t}$ *are caused by the same hidden factor* $M_{t}$*, whereas in a separate cause model (right box), different causes* $M_{t}$ *and* $V_{t}$ *are inferred for the proprioceptive* $P_{t}$ *vs. visual* $V_{t}$ *inputs. The switching node* $\boldsymbol{W}$ *compares the marginal log likelihood of the observations under a common- vs. separate cause assumption and determines the dominant model in each trial. The joint probability distribution of the network is given by the priors and the product of all transition- and emission factors. A delay is detected when the posterior marginal probability of the common cause model (CC) given the observations exceeds the marginal posterior of a separate cause (SC) model.*

- Furthermore, we provide a tabular overview of the parameters of interest that are optimized later on:
-

  Figure 3, l. 281

**R1.5**

Please insert the figures at the appropriate places in the manuscript. No journal is draconian enough to still want figures at the end.

- Done!

**R1.6**

L405 remove duplicate ‘for the’

- Done, thanks

**Reviewer 2**

Eckert and colleagures present a computational study to offer a novel explanation for the well-known phenomenon of sensory attenuation: The study develops a Bayesian causal inference (BCI) model to explain sensory attenuation of predictable sensory inputs because they were internally self-generated by motion (i.e. arise from a common signal cause as prioprioceptive signals) in contrast to externally attributed sensory inputs that are not attenuated because they inferred to come from an independent cause. The study develops the BCI model for two motion tasks and validates the BCI model on two previously published data sets using simulations and individual fits to the data. The authors report a decent fit of the model to the data and the model approximately fits important effects in the behavioral data.

While the theoretical contribution of the work is novel and important (i.e., explain sensory attenuation by BCI), I have many concerns about the computational modelling and presentation of results. Specifically, many details on the model formulation and fitting are lacking so that I doubt it would be possible to independently replicate the analyses (which I find especially important when introducing a novel model; see my comments below). Additionally, given the relatively few experimental conditions, the models appear highly complex with many free parameters whose meaning is unclear (i.e., difficult to interpret as indices of some cognitive mechanisms). Further, the manuscript needs some quality checks to increase the readability, especially on the figure and figure legends.

**R2.**

- We thank the reviewer for the generally positive evaluation and the fruitful suggestions for improvement.

My more specific comments:

**R2.1 Intro**

The intro provides three important accounts for sensory attenuation, i.e. efference copies, active inference and Bayesian causal inference. It may be a matter of style, but the links between the three accounts appears quite loose (e.g. which and how can shortcomings of efference copy account can be explained by active inference / BCI?).

- We re-formulated the paragraph introducing the different theoretical conceptualizations of sensory attenuation and decided to simplify the paragraph on the Active Inference framework. We particularly worked on the links between the theoretical frameworks.
- L. 66: *More recent theoretical developments extend optimal control theories and place many brain functions, such as perception, action and learning, under the mantle of Bayesian inference.*
- L. 81: *Similarly to Active Inference, Bayesian Causal Inference (BCI) is a normative framework of cognitive functions that centers around the idea of inferring the hidden causes of observations*
- We also make it clearer that we borrow concepts from both frameworks for our model:
- L. 97: *We here draw on the theoretical frameworks of Active Inference and BCI to develop a computational model of sensory attenuation.*

**R2.2**

**Methods**
Exp. 1 manipulation check: Did participants stroke with the targeted motion speed of 203mm/s that influences the intended frequency of stimulation? This might have been presented in the original publication, but should be referenced here.

- The participants performed several practice trials with visual feedback to ensure they adhere to the target motion speed. In addition, movement speed was recorded during the experiment using a force-feedback device. If participants deviated considerably from the target movement, the software repeated the respective trial and the experimenter gave oral feedback (see original publication). We also added this information into the methods section:
- L. 132*: Several practice trials with visual feedback were implemented to ensure participants adhere to the target motion speed.* *The empirical motion speed was 203 mm/s ± SD 35 mm/s (range: 110-329 mm/s, see original publication, supplementary Figure 6). If participants deviated considerably from the prescribed movement, the trial was repeated and verbal feedback was given.*
- Details on the average movement duration can be found in the original publication’s supplementary Fig. S6:
  <https://www.pnas.org/doi/suppl/10.1073/pnas.2118445119#supplementary-materials>

**R2.3**

In the Methods description of Exp. 2 and Fig. 1B the experimental factors are not consistently reported: The overall design manipulated 2 agency X 2 hand identity X video delay (0 – 417 ms)?

- Correct, we omitted the experimental manipulation of hand identity. We now elaborate on the reasons for this:
- L. 156: *We do not consider the experimental manipulation of hand identity here since it did not have a significant effect on behavior, with an insignificant main effect of hand identity on psychometric function threshold and slopes F(1,22)=0.185, p=0.671 and no interaction between hand identity and agency (active vs. passive), F(1,22)=0.744, p=0.332 (cmp. [6]).*
- We also updated the figure legend of figure 1B:
- *Note that we omitted the experimental manipulation of hand identity for simplicity (see Methods, Experiment 2). For more details, please refer to the original publication.*

**R2.4**

Justification and details of the BCI model:

Exp.1: It is unclear why the task is modelled with sequential BCI using an HMM: Even though the stimulus is extended in time, a “classical” BCI would assumed “accumulated / final” percepts for the frequency of haptic self-stimulation and the frequency of external tactile stimulation and compute the two potential causal structures from these likelihoods (and the prior; cf. Koerding 2007). Thus, it is unclear why the dynamics of stimulation are modelled here, especially when the behavioral response depends on a final “static” perceptual estimate. While modelling the dynamic might be interesting for future studies (e.g. EEG), this seems to add a lot of complexity here. This questions also holds for exp. 2.

- Our reasoning behind a sequential Bayesian graphical model was twofold.
- 1. Indeed, a sequential model would allow us to capture intra-trial dynamics that go beyond accumulated percepts, i.e. empirical patterns emerging from motion capture (Voudouris & Fiehler, 2021, *Sci Rep*) or EEG data (e.g. Kaiser & Schütz-Bosbach, 2018, *Eur J Neurosci*).
- 2. we borrowed several concepts from the Free Energy Principle and Active Inference, and a hierarchical, partially observable model is closest to the generative models typically used in this context (e.g. Parr, Pezzulo & Friston, 2022, *MIT Press;* Heins et al., 2022, *arXiv*). We make this clearer also in the introduction:
- L. 97: *We here draw on the theoretical frameworks of Active Inference and BCI to develop a computational model of sensory attenuation.*
- We now elaborate on our reasoning in the discussion section:
- L. 587: *Our model’s sequential nature offers potentially high temporal resolution of intra-trial dynamics, which are here inferred based on dichotomous behavioral responses. However, sensory attenuation shows interesting dynamical patterns in motion capture (28) and EEG (60) which we can account for in a sequentially structured model. The BCI model’s fit with these phenomena needs to be investigated in future studies.*

**R2.5**

Fig. 3B and text: It is unclear how “experimental manipulations were translated into a series of frequency observations”. From which model variables (e.g., likelihood distributions, probability estimates for external event) were the frequency observations derived? Fig. 3B axis labels suggest that frequency is changing as a function of time which was clearly not manipulated in the experiment…

- The frequencies were derived directly from what the participant observed in a trial. We extended our description of the conversion process and provide an example now:
- L. 216: *For qualitative simulations, we directly converted the sequence of frequencies within one trial into observations that were then input into the model’s observable layer (Figure 3B). For example, in a high-frequency congruent trial, the observed frequencies are converted into a list like (shortened)* $\left[ 0,0,0,30,0,0,0,30,30,30 \right],$ *where the first instance of a frequency of 30 (surrounded by zeros) represents the probe presentation, and a longer phase of observing the frequency represents object contact (padded to achieve a time series of length* $T=50$*).*

**R2.6**

The model parameters (e.g. mean and variance of likelihood distribution) and their meaning are not clearly described (e.g., in an overview). In exp. 2 there are seemingly 20 (!) parameters, and it is unclear where they come from exactly and what they mean. This is later partially mentioned in the results, but I think the reader should already get an overview of parameters in the methods.

- We introduced two new visualizations of the model and the parameters so that it is easier for the reader to understand their function in the factor graph:
- Figure 2, p. 14

- Figure 3, p. 15

- Note that we also updated the model’s architecture with the goal of reducing the number of free parameters to be optimized. We furthermore reduced the number of free parameters to be optimized for the model of Exp. 2 via softmaxing conditional probability distributions where applicable (previously 20, now 9), following your comments below.
- This point has also been raised by reviewer 1 (**R1.1**)

**R2.7**

H0 / H1 could be explained in more detail. H0 is a very weak competing model, i.e. uniform random choice of the three possible hidden states. Are there more powerful alternative models beyond causal inference (e.g., classically, BCI models are compared to forced-fusion and forced-segregation models)?

- In line with this suggestion, we have developed stronger comparison models for both Experiments 1 and 2.
- *Experiment 1*: The full causal model of Experiment 1 is now compared to a “bias” model, where the predicted response is a choice that is weighted by the individual’s idiosyncratic bias, in addition to the uninformative model. In the case of Experiment 1, a forced fusion/ forced separation approach did not seem to make a lot of sense to us due to the experimental design. The design here is quite far removed from a multisensory integration context where forced fusion/ separation models are typically applied. For this reason, we decided to implement a bias-specific model instead. The idiosyncratic biases turned out to explain a big proportion of response behavior and hence constitute a strong competitor model. However, the full causal model ended up with a small advantage over both the bias- and the uninformative model. The models were compared using the Laplace approximation to the posterior model evidence (LAP). LAP is a better approximation to the posterior model evidence than the BIC (Endres, Chiovetto & Giese, 2013). Furthermore, the LAP is a better choice to evaluate our model because the BIC is an approximation of the posterior model evidence in the limit of large datasets, which are not given here.
- We describe our model comparison in the article:
- Methods, L. 241: *We compared the full, causal model to two alternative models, a bias-only model and an uninformative model. In the bias-only model, participants are expected to show idiosyncratic response patterns in the probe detection task, which are not directly related to the experimental manipulation. The uninformative model predicts equal detection probabilities for all three causes (p=0.33). To compare the models* $M$*, we approximate the model evidence, or the log probability of the data* $D$*under the model and a tuple of its parameters* $\Theta$*:*

$$\log p\left( D | M \right)= \log\int d\Theta p\left( D | \Theta,M \right) p(\Theta|M)$$

*[5]*

*After evaluating the model evidence for all models, we compare model evidence differences. As usual in Bayesian approaches, the integral over model parameters is difficult to evaluate. We hence approximate the model evidence using the Laplace approximation LAP (58,60). The LAP is given by (60)*

$$p\left( D | M \right)\approx\underset{log-likelihood}{\underbrace{\log\left( p\left( D | \Theta^{*}, M \right) \right)}}+\underset{log-prior}{\underbrace{\log(p\left( \Theta^{*} | M \right))}+}\underset{log-posterior volume}{\underbrace{\frac{dim(\Theta)}{2}\log(2\pi)-\frac{1}{2}\log(\left| \mathbf{H} \right|)}}$$

*[6]*

*We evaluate the Hessian* $\mathbf{H}$ *with autograd [cite, v.] and find the optimal model parameters* $\Theta^{*}$*using the L-BFGS-B optimizer from the scipy.optimize library [cite, v.]*

*The LAP is a more accurate approximation to the model evidence than the BIC. Endres, Chiovetto and Giese (2013) show that the BIC performs comparably to the LAP only in the limit of datasets larger than ours. Therefore, LAP is a more exact approximation to the Bayesian model evidence than the BIC in the present case.*

- Results, L. 395: *Model comparisons yielded an advantage for the full causal model. The evidence difference per participant (∆ LAP causal – LAP bias) was 1.20 (± standard error 0.15), indicating an advantage for the full causal model over a bias-only model on the participant level. This translates into a probability ratio of 4.9x10^16on the group level. This indicates strong superiority of the causal model on the group level. The bias-only model held an advantage over the uniform model (∆ LAP bias - LAP uniform), with a group-level average of 28.54 ± standard error 5.46 (Figure 8).*

Figure 8. Model comparison, Experiment 1.

*The full causal model is compared to a model where responses are predicted exclusively by individual biases (bias) and one with uninformative parameters (uniform). Displayed are probability rations, or differences (*$\Delta$*) in LAP. Positive values indicate an advantage for the causal model compared to the bias-only model, and an advantage over the bias model over a uniform model. Black lines represent standard errors.*

- *Experiment 2*: For Experiment 2, we implemented the classical comparator models of forced fusion and forced separation. All models were compared using the BIC. In the forced fusion model, only the common cause model is used to fit the data, which translates to a simulated probability of a separate cause that is equal to p(sep)=0. In the forced separation model, only the separate causes model is used to fit the data, which corresponds to a simulated probability of separate causes p(sep)=1. To avoid under/overflows, we use p(sep)=0.01 and p(sep)=0.99 respectively. We also fitted a bias model as in experiment 1 as a baseline.
- For both experiments, all comparator models, the bias model and the full causal model were evaluated using the Laplace Approximation as before.
- Methods L. 346: *We compare the full causal model to two competing models commonly used in multisensory integration research: forced fusion and forced separation (50,57). In a forced fusion model, the proprioceptive and visual information will always be attributed to the same hidden cause. Contrarily, in forced separation, the visual- and proprioceptive information streams are always attributed to separate underlying causes.* *We furthermore introduce a bias-only model as before, where participant choices are a function of their idiosyncratic response biases. The Bayesian model evidence is approximated using the LAP, as described previously (58,63).*
- Results*:* L. 427*. A model comparison yielded an advantage for the full causal model over all competing models, namely, a bias model, a forced fusion- and a forced separation model. The superiority of the full causal model held in both active- and passive movement trials (Figure 13). The evidence difference per participant (∆ LAP causal – LAP bias) in active movement trials was 18.82 (± standard error 1.98), indicating an advantage for the full causal model over a bias-only model on the participant level. The advantage approached certainty on the group level (1.62e^196) The bias-only model held an advantage over the forced fusion model (∆ LAP fusion - LAP bias -101.80 ± 10.06). The bias model was superior to a forced separation model (∆ LAP separation - LAP bias: -124.87 ± 11.15). In passive movement trials, the causal model was superior to a bias model (∆ LAP causal – LAP bias: 21.07±1.73). The bias model was superior to a forced fusion model (∆ LAP fusion – LAP bias: -125.77±10.54) and to a forced separation model (∆ LAP separation – LAP bias: -100.08±9.76).*
- Results Figure:

Figure 13. Model comparison, Experiment 2

***A.*** *Model comparison on active movement trials: a full causal model is compared to a bias-only model (causal-bias). Positive values indicate a higher model evidence for the full causal model. The bias model is superior to a forced fusion model (fusion-bias), and a forced separation model (separation-bias).*

***B.*** *Model comparison on passive movement trials. The full causal model is superior to a bias-only model. The bias-only model is superior to a forced-fusion and a forced-separation model.*

**R2.8**

Overall, details are missing how the generative model in exp. 1 and 2 is linked to inferred internal states/estimates and is finally linked to behavioral responses (e.g. how is p(ext) finally estimated? What is exactly the likelihood function in exp. 2?)

- We hope that our updated Figures 2 and 3 now give sufficient details about this matter.
- Specifically, we included the computation to transform model quantities into behavioral responses in Figure 2 (“Detection response”).
- For Experiment 1, this is given by $p\left( M_{12}=ext \right)>p(M_{12}=int,none)$: a detection response occurs when the posterior probability of an external cause exceeds the posterior probability of an internal (or none) cause at timepoint 12, when the probe is presented in the time series.
- For Experiment 2, this is given by $p\left( SC | o \right)>p(CC|o)$: A detection response occurs when the conditional probability of a separate cause model given the data (p(SC|o)) exceeds the conditional probability of the common cause model (CC) given the data.
- This issue is also further elaborated on in the figure legends and in the text:
- Fig. legend, l. 261: ***A.*** *Graphical model for Experiment 1. Hidden nodes M_t (unfilled circles) form a Markov chain that can take on values {internal, external, none}. Observable nodes F_t (filled circles) can process frequency inputs between 0-100 Hz. The joint probability distribution over the network is given by the prior and the product of all transition- and emission nodes. A detection response (r_t=1) occurs when the normalized posterior probability of the external cause exceeds the normalized posterior probability of an internal or none cause.* ***B.*** *Graphical model for Experiment 2. Under a common cause model (left boxtop), proprioceptive P_t and visual information V_t are caused by the same hidden factor M_t, whereas in a separate cause model (right boxbottom), different causes M_t and V_t are inferred for the proprioceptive P_t vs. visual V_t inputs. The switching node W compares the marginal log likelihood of the observations under a common- vs. separate cause assumption and determines the dominant model in each trial. The joint probability distribution of the network is given by the priors and the product of all transition- and emission factors. A delay is detected when the posterior marginal probability of the common cause model (CC) given the observations exceeds the marginal posterior of a separate cause (SC) model.*

**R2.9**

Was model fitting in Exp. 1 repeated with multiple parameter initializations to check stability of the results (in exp. 2 at least 2 initializations)?

- Yes, the optimization was performed with jittered starting values 10 times for each participant. For each participant dataset, the best set of parameters (i.e., the set that minimized the cost) among the 10 options was chosen. We now mention this more explicitly in the methods section:
- L. 234: *For each participant, the optimization was initiated 10 times, where the optimizer’s starting values were jittered within the predefined bounds for each optimization to ensure the results’ robustness. The best set of parameters (i.e., the parameters minimizing cost) was chosen for each participant.*
- L. 336: *For each participant dataset, the optimization was initiated 10 times and the best resulting set of parameters was selected (i.e., the parameters that minimize cost across the 10 results).*
- Please note that for reasons of simplicity, we removed the reference to the two different runs of the optimization for Experiment 2, which we omitted after re-instating the model with a reduced number of parameters.

**R2.10**

It is actually good practice in computational modelling (e.g. Wilson & Collins, 2019) to perform parameter recovery (and even model recovery) to show that the parameter are reliably estimated with the used fitting procedure. Especially for a novel computational model, this appears to be important.

- Of course, the reviewer is right in suggesting that a parameter recovery is important for validating our model. We have performed a parameter recovery and added our insights into the revised version of the manuscript. This point has also been raised by reviewer 1 (**R1.2**)
- The parameter recovery for the two experiments followed a similar logic:
  - **Experiment 1**:

1. initialize subject-specific model with best parameters obtained from the optimization, and obtain sampled probability for the hidden causes (external, internal, none).
2. Make a weighted random choice between 1 (detection of probe) and 0 (no detection), where the weights are provided by the model-predicted p(external) (for a 1 response) and the model-predicted p(internal)+p(none) (for a 0 response).
3. Build dataset with sampled responses
4. Perform optimization on the sampled data per participant and obtain subject-specific parameters based on sampled data
5. Compare the subject-specific parameters optimized on sampled data with the subject-specific parameters obtained from the optimization on the empirical data.
   - **Experiment 2:**
1. Initialize subject-specific model with parameters obtained from optimization on empirical data and obtain sampled marginal probability of a common vs. separate cause model.
2. Make a weighted random choice based on the marginal probabilities of each model, where a “1” choice refers to a delay detection and is weighted by the marginal probability of separate causes, and a “0” represents a non-detection and is weighted by the marginal probability of the common cause model.
3. Build dataset with sampled responses
4. Perform optimization on the sampled data per participant and obtain subject-specific parameters based on sampled data
5. Compare the subject-specific parameters optimized on sampled data with the subject-specific parameters obtained from the optimization on the empirical data.

- Results for Experiment 1 recovery suggest a good agreement between simulated and empirical parameters:

Figure S14. Parameter recovery, Experiment 1.

***A.*** *Empirical- and simulated parameters for Experiment 1 data. Black distributions represent group-level, averaged parameters across 5 runs of the optimization on simulated data. Blue stars represent the mean (with vertical error bars), group-level parameters obtained from optimization on empirical data.*

***B.*** *Motor noise parameter, obtained from empirical data (blue distribution) vs. from simulated data (red distribution).*

- These results are mentioned in the main part of the manuscript and in more detail in the supplementary materials S2.

Figure S15. Parameter recovery, Experiment 2.

**A.** Parameter recovery for active trials of Experiment 2. Grey violins are recovered parameter distributions, blue stars represent the mean ground truth parameter, and blue lines are standard deviations. **B.** Parameter recovery for passive trials of Experiment 2.

**R2.11**

In exp. 2, the model is separately fitted to active vs. passive movement, I assume by refitting all 20 model parameters. This effectively doubles the number of parameters to fit a single experimental observations, which is not a very sparse way to explain the effects. Is the distinction active vs. passive not better captured by a single parameter with a meaning (e.g. a sensory variance)?

- This point is well taken, the model for Exp. 2 is very complex and in the revision, we tried to simplify it and reduce the number of parameters for the optimization. This is achieved in the following ways:

1. We reduced the number of parameters by computing (conditional) distributions by softmax, with the last entry fixed at 0.
2. We now assume the same likelihood model between the common- and separate cause model. This assumes that perceptual processing of observations caused by common- vs. separate causes is the same and reduces the number of parameters to optimize further. In the new model, we are hence working with 9 instead of 20 parameters.

- The graphical model is now updated (compare Figure 2), where ***W*** is the switching node that compares between common- and separate cause log marginal likelihood and chooses one after observations.

- The more reduced model results in similar qualitative results and improved quantitative predictions, see Figure 11 (p. 29)
-

**R2.12**

Fig. 3C: x/y axis legends missing.

- Updated, thank you:
-

  ***C.*** *Observation schemes per trial type, non-delay trials (left), where visual and proprioceptive inputs follow the same temporal pattern (i.e., shifting in state at the same time), vs. delayed trials, in which the visual information is shifted against the proprioceptive input in time.*

**Results**
**R2.13**

Fig. 4: Why does the attribution of internal/external cause appears only transiently, but after “object contact” always shifts to internal? From figure 6 I can learn that around this time the probe is presented, but how does this affect the model when the haptic signal is actually only presented later (after object contact)? This needs better explanation.

- We have tried to address this point by elaborating on the explanations of these findings in the results section:
- L. 370: *This pattern is mediated by the nature of the probability distribution contained in the emission nodes, where a given frequency that is expected under the current motion policy determines the mean of a normal distribution. When the probe’s frequency matches this mean expectation, an internal cause for it is inferred. In the incongruent case, the mean expected frequency is different from the one experienced during probe presentation, and hence an external cause is inferred for it. Under an external cause, all frequencies are equally (un)likely, which is why the model prefers it for unexpected frequencies.*

**R2.14**

(Please also check legends A, B, C – assignment wrong).

- Corrected:
- L. 356: ***A.*** *Simulation of a congruent trial, where an internal cause is inferred for the probe.* ***B.*** *In the incongruent probe trial, an external cause is inferred for the probe.* ***C.*** *In the trial without probe, no external or internal cause is inferred.*

**R2.15**

Fig. 5A: The model’s replication of the behavioral congruency effects (which also appear rather weak) is not very good – how good is the overall model fit (e.g. explained variance / coefficient of determination)?

- We now approximate Bayesian model evidence with a Laplace approximation and report the model to a comparator model that consisted only of idiosyncratic response biases.
- We also discuss the rather small behavioral effect in Experiment 1 and the implications for our model in the discussion section:
- L. 535: *Note that the model’s ability to capture these behavioral effects might be due to relatively small empirical differences between the experimental conditions. To best test our model in the context of tactile sensory attenuation, it may require larger differences between experimental conditions on the behavioral level.*

**R2.16**

The results present first simulations, then partially individual results with individual parameter fits. It is unclear why not simply average results at group level are presented with individually fit parameters – that would increase the readability of the manuscript.

- Thank you for pointing this out to us – we now report group-level results for Experiment 1 instead of one example participant.
- Figure 7, p.25:
-

-

**R2.17**

Bernoulli factors in exp. 1 and 2: Bernoulli factor compare the fitted model to a non-fitted model with fixed parameters, but do Bernoulli factors take model complexity into account? E.g. Bayesian information criterion penalizes free parameters when comparing the fit of two models…and the null model has 0 and the main model has 12 / 20 (?) free parameters. I.e. the null model needs a fairer comparison, and the author might consider a stronger comparison model (i.e. forced fusion /segregation)

- We have deleted the results on the Bernoulli factors and performed a model comparison with stronger competing models (as suggested above) on the basis of the Laplace Approximation, which takes model complexity into account.

**R2.18**

I have a hard time to match Figure 8A and its legend…what is the figure showing exactly? PSE and relative model evidences?

- Correct, the 8A shows the PSE as a horizontal, red dashed line, and the respective model evidences for the common and separate cause models as a red and green line. We updated the legend to make it more understandable
- L. 478: *The simulated point of subjective equality (PSE)is represented by the red dashed line, indicating the PSE is located at a delay of 5 ds. In smaller delays, the common cause model (coral line) has a higher marginal probability. For larger delays, the separate cause model (turquoise line) is inferred as more likely.*

**R2.19**

Model reproduction of the threshold-shift for active vs. passive movement: As stated in the comment above, it appears highly unsparse to refit a full set of parameters to explain a single experimental manipulation. As a result, the effect of the manipulation cannot be attributed meaningfully to parameters (Supplemental fig. 16). The authors might consider which of the model’s parameters is likely affected by an active/passive manipulation, and more systematically analyse which parameter can explain the effect (e.g. priors vs. sensory variances).

- We have reformulated the model as suggested, with the observation- and action model sharing the likelihood factor in both models. This has improved our fitting results.
- Before: (collapsed across active/ passive trials)

- After:

- An experimental manipulation of the model’s parameters is a highly interesting idea, which we refer to in the discussion section now:
- L. 593: *For instance, experimental work is necessary to study how precisely model parameters are affected by specific experimental manipulations.*

**R2.20**

Fig. 12: Again, it should be made clearer that A is participant data and B is model prediction. It is unclear why 6 participants are removed from B, appears somewhat as an adhoc choice.

- In the revised version of the model, there were no more absolute misfits – as previously, there were 6 datasets that were not captured by the model in sufficient quality. This problem has been solved with the proposed update of the model for Experiment 2.
- We report group-level data only, and updated figure 13:
- L. 503,
-

**Discussion**:

**R2.21**
I agree that the developed BCI models would be an attractive option to quantify psychopathological abberations e.g. in schizophrenia, but I would add the caveat that the model first needs more basic testing and experiments before such an effort is fruitful (e.g. parameter recovery as a start, test-retest reliability of parameters, specific experiments to explore which experimental manipulations affect which parameters etc). The models are an important starting point, but they are also very complex (i.e., many parameters and estimation of dynamics) and need more validation in future studies.

- An important point – we added this in the discussion.
- L. 586: *The present work provides a starting point for computational descriptions of sensory attenuation. […] While the model passed initial validation procedures, future effort needs to be directed towards thorough testing and empirical validation of our model.* *For instance, experimental work is necessary to study how precisely model parameters are affected by specific experimental manipulations. The test-retest reliability of individual-level parameters across time and across experiments needs to be established. If successful, the BCI model could be applied to study altered sensory attenuation in clinical populations. As previously mentioned, failures of sensory attenuation have been connected to false inferences about agency and body ownership in schizophrenia (32,34–37). In future efforts, our computational model may be expanded towards formalizing aberrant Causal Inference in patients with psychosis or heightened levels of psychosis proneness.*
